# Sustainability in Higher Education: Experience report on achieving Sustainable Development Goals

**DOI:** 10.1590/0034-7167-2024-0118

**Published:** 2025-01-10

**Authors:** Laura Mafalda Carvalho Lopes, Rosalina Matias-Correia, Rosa Carla Gomes da Silva, Natália de Jesus Barbosa Machado, Ana Paula dos Santos Jesus Marques França

**Affiliations:** I Escola Superior de Enfermagem do Porto. Porto, Portugal; II Centro de Investigação em Tecnologias e Serviços de Saúde. Porto, Portugal

**Keywords:** Sustainable Development Indicators, Universities, Social Responsibility, Nursing, Agenda 2030, Indicadores del Desarrollo Sostenible, Universidades, Responsabilidad Social, Enfermería, Agenda 2030

## Abstract

**Objective:**

Describe the activities aimed at achieving the Sustainable Development Goals (SDGs), implemented by a polytechnic higher education institution, in the northern region of Portugal.

**Methods:**

This is an experience report from a Higher Education Institution, which characterizes the contribution to the SDGs, within the scope of teaching, research, campus and leadership.

**Results:**

In total, 1247 activities were mapped, with a preponderance in the “people” dimension (685 activities), with SDG 3 being the most prominent. Scientific articles contribute significantly to the SDGs, with a notable discrepancy between automatic (Scopus) and manual classifications, indicating a tendency to underestimate the impact of nursing studies.

**Conclusion:**

The results demonstrate the significant contribution of this institution to the SDGs, with a special focus on health and education. This contribution reflects the mission of the institution, which involves training competent and socially conscious health professionals, but simultaneously the need for greater awareness and training of the teaching staff.

## RESUMEN

**Objetivo:**

Describir las actividades de la concretización de los Objetivos del Desarrollo Sostenible (ODS), implantadas por una institución de educación superior politécnico del norte de Portugal.

**Método:**

Se trata de un relato de experiencia de una Institución de Educación Superior, que caracteriza la aportación para los ODS, en la esfera de la enseñanza, la investigación, el campus y el liderazgo.

**Resultados:**

En total, se mapearon 1247 actividades con preponderancia en la dimensión “personas” (685 actividades), destacándose el ODS 3. Los artículos científicos contribuyen significativamente a los ODS, con una notable discrepancia entre las clasificaciones automática (Scopus) y manual, lo que indica una tendencia a subestimar el impacto de los estudios de enfermería.

**Conclusión:**

Los resultados demuestran la contribución significativa de esta institución a los ODS, con énfasis en la salud y en la educación. Esto refleja la misión de formar profesionales sanitarios competentes y con conciencia social y al mismo tiempo, la necesidad de sensibilizar y capacitar al cuerpo docente.

## INTRODUCTION

In the current situation, the search for sustainable and socially responsible solutions emerges in different spheres of society. Higher Education Institutions (HEIs) play a crucial role in the development of future societies, not only by training qualified professionals, but also by promoting innovative leadership. From this perspective, these institutions are called upon to take on the responsibility of incorporating the Sustainable Development Goals (SDGs) into their teaching, research and extension activities, as well as into their strategic plans^(1)^.

The United Nations (UN) 2030 Agenda, established in 2015, commits 193 countries to seeking sustainable development through 17 SDGs. These SDGs cover the economic, social and environmental dimensions, aiming to balance global growth. The SDGs define sustainability goals in areas considered deficient and are structured into five principles (5P) - Planet, People, Prosperity, Peace and Partnerships - which guide the search for a sustainable future. Each principle has specific goals such as protecting the planet, eradicating poverty, promoting prosperity, ensuring peace and building global partnerships for sustainable development. This agenda represents a global commitment to face urgent challenges, such as climate change, poverty and hunger, emphasizing global solidarity and the inclusion of everyone in the search for a more sustainable world^(2)^.

These principles, that is, the 5P, reflect a holistic and integrated vision of sustainable development, emphasizing the need for joint and collaborative actions between countries. Thus, the SDGs emerge as a roadmap for a sustainable future, guiding nations around the world in the search for solutions that balance economic, social and environmental needs, while simultaneously facing global challenges, from social inequality to climate change^(2)^.

The term “sustainability” has roots in the Latin word sustainable, referring to the state of maintaining something at a certain level, degree or value^(3)^. The concept of sustainable development gained international recognition in 1972, at the United Nations Conference on the Human Environment in Stockholm, which addressed the harmful impact of the indiscriminate use of natural resources. This concept was solidified in 1983, when the World Commission for Environment and Development produced the Brundtland report, highlighting that sustainable development must meet present needs without compromising the ability of future generations to meet their own needs, allowing social development to be achieved, economic and human satisfaction, making rational use of resources and preserving nature^(4)^.

Rio de Janeiro’s Summit, in 1992, sought to reconcile economic development with environmental protection, resulting in Agenda XXI^(5)^. The concept of sustainable development was highlighted again in 1998 during the World Conference on Higher Education in the 21^st^ Century - Vision and Action, which took place in Paris. In 2005, the UN declared the decade of education for sustainable development, aiming to change behaviors and empower populations to build a more sustainable world. Finally, in 2015, the 17 SDGs were included in the 2030 Agenda, with the aim of promoting the acquisition of knowledge and skills for sustainable development. Leal-Filho *et al*. (2017) state that the SDGs can constitute an opportunity to overcome obstacles to sustainability in HEIs^(6)^. Although the study by Aleixo *et al*. (2018) suggest that HEIs and society recognize the great importance of sustainable development, this has not yet been fully integrated into the system and institutional activities^(7)^.

It is important to assess the extent to which HEIs incorporated sustainable development in all their activities, which generally followed a “top-down” approach. This process began with initiatives planned by management bodies, subsequently involving all interested parties. A sustainable university, according to Velazquez *et al.* (2006), is an HEI that, in part or as a whole, addresses, involves and promotes the minimization of negative impacts on the environment, economy, society and health, occurring through the fulfillment of its functions of teaching, research, dissemination, partnership and management, contributing to the transition to a society with more sustainable lifestyles^(8)^.

According to Ramos *et al.* (2015), the development of higher education for sustainability implies a more effective inclusion of education for sustainable development in courses. Although case studies documented in recent years demonstrate changes in HEI curricula, education for sustainable development is still not widely practiced and remains a significant challenge that needs to be met^(9)^.

HEIs play a fundamental role in promoting sustainable development, thus contributing significantly to the SDGs. This contribution occurs in four essential areas, aligned with the mission and activities of the HEIs. These areas include conducting innovative research, offering teaching aligned with sustainability principles, responsible environmental management of campuses and active leadership in promoting sustainable practices^(10)^.

This study therefore focuses on the role of HEIs in achieving the SDGs, exploring how they can integrate them into their daily activities, such as academic curricula, research activities, campus and leadership. When considering institutional commitment to the SDGs, we will examine an example of a Portuguese HEI that has been a catalyst for significant changes towards more sustainable development.

## OBJECTIVE

To describe the activities in the areas of Research, Teaching, Campus and Leadership, which aim to achieve the Sustainable Development Goals (SDGs), promoted by a Higher Education Institution, integrated into polytechnic education, in the northern region of Portugal.

## METHODS

This is an experience report from an HEI, during the year 2022, regarding activities aimed at fulfilling the 17 SDGs^(10)^ in different types of activities.

### Experience report context

In 2021, this HEI joined the Observatory of Social Responsibility and Higher Education Institutions (ORSIES), which resulted in the mapping of activities in the area of teaching and research that comply with the SDGs.

This HEI, which exclusively teaches nursing, has the vision of building more meaningful nursing for people. In this sense, it establishes a commitment to society, the profession and the community to build knowledge-based Nursing and encourage the acquisition of skills that respond to the societal challenges that the 21^st^ century imposes (Strategic Plan 2020-2024).

According to the guide “Accelerating education for the SDGs in universities: a guide for universities, colleges and higher education institutions”^(11)^, HEIs can contribute to the SDGs in four areas, considering their mission and intervention, namely, research, teaching, leadership and management.

### Procedure for collecting and organizing information

Recognizing the importance of sustainability practices, the indicators that respond to the SDGs were analyzed, through the mapping and classification of activities according to the recommendations of SDSN Australia^(1)^. This mapping was carried out using a manual process of reading and interpreting information. This approach involved a documentary analysis of data sources and a manual assessment, with the respective attribution of activities to the SDGs, as it is technically and logistically a simpler approach.

**Figure 1:**
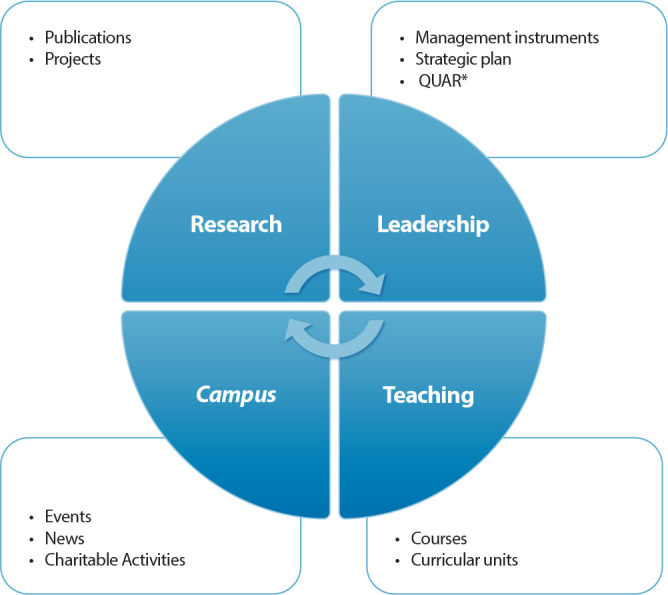
Activities considered in mapping by area

Legend: *QUAR - Assessment and accountability framework.

In order to organize the information, the results mapped by SDG, from the activities carried out at this HEI, were grouped into the following categories: teaching, research, campus and leadership (for more details see Figure 1). More specifically, in the teaching category, there are different courses taught by this HEI and the curricular units (CU) of the Nursing degree course (NDC). For this purpose, the sources of information subject to analysis were the NDC CU sheets and global information from the remaining courses; in the research, publications of scientific articles were mapped, through reading the titles and abstracts, as well as the analysis of ongoing research projects; Already on campus are activities that give “life to an academic campus” such as events (e.g. congresses), news of events held and promoted by this community, podcasts and also activities of a charitable nature; finally, leadership activities such as management instruments in use, strategic plans and the Assessment and Accountability Framework (AAF). AAF is a reference framework for evaluating the performance of services (mission), their action purposes (strategic objectives), the goals to be achieved, the performance indicators and respective sources of verification, the available resources (human and financial) and the measurement of its implementation and the summary identification of deviations and their respective causes determined at the end of the management cycle. The data generated was analyzed using descriptive statistics, using tables and graphs to summarize the information.

## RESULTS

Figure 1 was adapted for mapping by areas (research, leadership, campus and teaching) and presents the activities considered in the organization of information.

Next, the results of the mapping are presented, for each SDG of the activities carried out at the HEI under study, referring to the areas of teaching, research, campus and leadership.

In the teaching area, the activities that contribute to the SDGs were quantified in relation to courses and UCs, see Table 1. Similarly, in the research area, the contribution of scientific articles and research projects under development by SDGs was identified. Furthermore, in Table 1, the campus area is highlighted, which comprises the number of events, news, podcasts and activities carried out in a solidarity context, and finally, it is possible to check the activities carried out within the scope of leadership that also contribute to the SDGs.

**Table 1:** Total quantification of 2022 activities that contributed to the SDGs

Courses	Courses	1	2	3	4	5	6	7	8	9	10	11	12	13	14	15	16	17
Teaching	Courses	1	0	33	33	0	0	0	1	33	0	0	0	0	0	0	0	0
	Curicular units	10	0	34	34	0	0	0	10	1	0	0	0	0	0	0	0	0
Research	Scientific articles	6	0	165	23	2	0	0	10	51	7	1	0	1	0	0	7	2
	Investigation projects	0	0	58	19	0	0	0	10	58	0	0	0	0	0	0	2	10
*Campus*	Events	2	0	8	18	1	0	0	2	1	3	1	0	0	0	0	14	13
	News	5	0	32	27	0	0	0	2	11	13	2	0	3	1	1	13	39
	Podcast	0	0	18	4	0	0	0	7	0	1	1	0	0	0	0	1	1
	Charitable Activities	23	17	42	33	8	11	5	5	5	49	5	9	5	5	5	54	48
Lidearship	Management instruments/Strategic plan / AAF	4	1	2	8	3	0	0	2	4	7	0	1	0	1	1	12	5

*Legend: AAF - Assessment and accountability framework.*

Regarding the teaching area (courses), Table 2 shows the distribution of SDGs by degree, master’s degree, postgraduate degree and doctorate.

**Table 2:** Attribution of SDG, for degree-awarding and non-degree-awarding courses, in 2022

Course/SDG	1	2	3	4	5	6	7	8	9	10	11	12	13	14	15	16	17
Undergraduate	1	0	1	1	0	0	0	1	1	0	0	0	0	0	0	0	0
Master’s degree	0	0	21	21	0	0	0	0	21	0	0	0	0	0	0	0	0
postgraduation	0	0	10	10	0	0	0	0	10	0	0	0	0	0	0	0	0
PhD	0	0	1	1	0	0	0	0	1	0	0	0	0	0	0	0	0
Total courses (undergraduate, master's, postgraduate and doctoral degrees)	1	0	33	33	0	0	0	1	33	0	0	0	0	0	0	0	0

With regard to scientific articles, a comparative analysis was made of the distribution of the SDGs assigned manually and by the Scopus classification as presented in Table 3. The manual assignment of the SDGs was carried out according to the content of the articles (title and summary) and not only by keyword, as in Scopus.

**Table 3:** Number of scientific articles distributed by the SDGs by manual classification vs Scopus classification

Classification	1	2	3	4	5	6	7	8	9	10	11	12	13	14	15	16	17
Manual	6	0	165	23	2	0	0	10	51	7	1	0	1	0	0	7	2
Scopus	1	0	12	0	1	0	0	1	1	2	0	0	0	0	0	0	0

Table 4 aggregates the total distribution of activities carried out in this HEI, by SDG and 5P, highlighting a total of 1247 activities.

**Table 4:** Total distribution of 2022 activities by SDG and 5P

ODS	People							Planet				Prosperity					Peace	Partnership	TOTAL
	1	2	3	4	5	6	7		8	9	10	11	12	13	14	15	16	17	
Atividades/contribuições	51	18	392	199	14	11	5		49	164	80	10	10	9	7	7	103	118	1247

*Legend: SDG - Sustainable development goals*

The data demonstrate that the IES, out of a total of 1247 activities mapped and classified for the SDGs, attributes greater preponderance to the “People” dimension, representing 55% (n= 685) of the classified activities. In the “People” dimension, emphasis is placed on the centrality of SDG 3 - Health and Wellbeing - which corresponds to 32% (n= 392) of the total activities listed.

Regarding the “Planet” dimension, with a total of 293 activities (24% of total activities), SDG 9 - Industry, Innovation and infrastructure stands out with 164 activities (13% of total activities).

In the “Prosperity” dimension (n=43), SDG 11 - Sustainable Cities and Communities and SDG 12 - Responsible Consumption and Production, present 10 activities each, corresponding to the residual part of the sample (3%). Finally, the “Peace” and “Partnerships” dimensions with 103 and 118 activities, each, representing 8% and 10% of the total activities classified.

## DISCUSSION

This HEI, which is focused on teaching and research in nursing, makes a contribution to the SDGs in the area of health and education, reflecting its mission to train health professionals and promote scientific knowledge.

In the context of teaching, the quantitative analysis of educational activities, such as courses and UCs, reveals a centrality for SDGs 3 (Health and Wellbeing), 4 (Quality Education) and 9 (Industry, Innovation and Infrastructure), with greater incidence in the first and second cycles of studies. However, there is an under-representation of other important SDGs, which may not correspond to the reality of the content taught, pointing to the need to raise awareness among teachers to intervene in the classification process.

In the research domain, the discrepancy between automatic classifications, such as those from Scopus (based on an algorithm that combines a set of keywords), and manual analyzes indicates a tendency to underestimate the contribution of nursing studies to the SDGs. Since the automatic classification does not seem to have reflected the impact of the articles in the area of global health and quality education, the manual analysis reveals a substantial contribution, especially for SDG 9 (Innovation). Therefore, in the case of publications in the area of Nursing, the SDG count according to Scopus does not seem to consider the contribution of this discipline to sustainability and social responsibility. Furthermore, this institution has a clear commitment to sustainability and social responsibility, demonstrated in its Strategic Plan 2020-2024, which presents actions aimed at health, safety, energy efficiency and community well-being. The institution is also committed to combating school dropout rates, as well as promoting the employability of its graduates, promoting inclusive education and practices that balance personal, family and professional life.

Solidarity activities and social responsibility initiatives have given dynamics and meaning to the campus, highlighting the commitment of this HEI to creating an educational environment that meets the needs of students, but also contributes to the sustainable development of society. Therefore, this experience report reflects work on monitoring, mapping and classifying activities by SDG, through a holistic approach that integrates teaching, research and social responsibility, capable of being replicated in other HEIs.

## CONCLUSION

In conclusion, data analysis highlights this HEI’s commitment to the SDGs, particularly in the areas of health, education and innovation.

This commitment is not only manifested in current activities, but also, in terms of implications for practice, in the strategic projection and implementation of specific sustainability indicators. From this perspective, this work has also contributed to the adoption and recording of indicators such as the number of students with financial support (SDG1), water consumption (SDG6), the number of patents (SDG9), among others. This reflects its systematically integrated strategy that aims not only to comply with the SDGs, but also to continuously monitor and evaluate the impact of actions on the institution. This practice facilitates continuous self-assessment, reinforces alignment with the institution’s strategic plan, ensuring that ongoing and future activities remain focused on promoting sustainable development. The integration of these indicators into the daily practice of the HEI reinforces its role as a leading institution in nursing education, not only in the northern region of Portugal, but also as a model of sustainability in the context of higher education. This strategic approach not only meets the immediate objective of meeting the SDGs, but also establishes a sustainable path for the future, where education, research and leadership intertwine to promote positive and lasting societal impact. Finally, it is concluded that there is a need for greater awareness and training of teachers responsible for planning academic content, in order to structure their CU or Courses, based on contributions to the SDGs.
