# Supplementary material for: Sustainability in Higher Education: Experience report on achieving Sustainable Development Goals
Source: Rev Bras Enferm. 2025 Jan 10;77(Suppl 2):e20240118. doi: 10.1590/0034-7167-2024-0118 (PMC11726806; doi:10.1590/0034-7167-2024-0118)
Supplement: Translation

# Translation: Sustentabilidade no Ensino Superior: Relato de experiência sobre o cumprimento dos Objetivos de Desenvolvimento Sustentável

**DOI:** 10.1590/0034-7167-2024-0118pt

**Authors:** Laura Mafalda Carvalho Lopes, Rosalina Matias-Correia, Rosa Carla Gomes da Silva, Natália de Jesus Barbosa Machado, Ana Paula dos Santos Jesus Marques França

**Affiliations:** I Escola Superior de Enfermagem do Porto. Porto, Portugal; II Centro de Investigação em Tecnologias e Serviços de Saúde. Porto, Portugal

**Keywords:** Indicadores de Desenvolvimento Sustentável, Universidades, Responsabilidade Social, Enfermagem, Agenda 2030

## Abstract

**Objetivo:**

Descrever as atividades que visam o cumprimento dos Objetivos de Desenvolvimento Sustentável (ODS), implementadas por uma instituição de ensino superior politécnico, da região norte de Portugal.

**Métodos:**

Trata-se de um relato de experiência de uma Instituição de Ensino Superior, que caracteriza o contributo para os ODS, no âmbito do ensino, investigação, campus e liderança.

**Resultados:**

No total, foram mapeadas 1247 atividades, com uma preponderância na dimensão “pessoas” (685 atividades), sendo o ODS 3 o mais destacado. Os artigos científicos contribuem de forma significativa para os ODS, com uma discrepância notável entre as classificações automáticas (Scopus) e manuais, indicando uma tendência de subestimação do impacto dos estudos de enfermagem.

**Conclusão:**

Os resultados demostram a contribuição significativa desta instituição para os ODS, com um foco especial na saúde e educação. Esta contribuição reflete a missão da instituição que passa por formar profissionais de saúde competentes e socialmente conscientes, mas simultaneamente a necessidade de maior sensibilização e formação do corpo docente.

## INTRODUÇÃO

Na conjuntura atual, a procura de soluções sustentáveis e socialmente responsáveis emergem em diversas esferas da sociedade. As Instituições de Ensino Superior (IES) desempenham um papel crucial no desenvolvimento das sociedades futuras, não apenas pela formação de profissionais qualificados, mas também pela promoção de lideranças inovadoras. Sob esta perspetiva, estas instituições são convocadas a assumir a responsabilidade de incorporar os Objetivos de Desenvolvimento Sustentável (ODS) nas suas atividades de ensino, investigação e extensão, bem como ao nível dos seus planos estratégicos^(1)^.

A Agenda 2030 da Organização das Nações Unidas (ONU), estabelecida em 2015, compromete 193 países a procurar o desenvolvimento sustentável por meio de 17 ODS. Esses ODS abrangem as dimensões económica, social e ambiental, visando equilibrar o crescimento global. Os ODS definem metas de sustentabilidade nas áreas consideradas deficitárias e estão estruturados em cinco princípios (5P) - Planeta, Pessoas, Prosperidade, Paz e Parcerias - que orientam a procura por um futuro sustentável. Cada princípio tem metas específicas como proteger o planeta, erradicar a pobreza, promover a prosperidade, garantir paz e construir parcerias globais para o desenvolvimento sustentável. Esta agenda representa um compromisso global para enfrentar desafios urgentes, como mudanças climáticas, pobreza e fome, enfatizando a solidariedade global e a inclusão de todos na procura de um mundo mais sustentável^(2)^.

Estes princípios, ou seja, os 5P, refletem uma visão holística e integrada do desenvolvimento sustentável, enfatizando a necessidade de ações conjuntas e colaborativas entre os países. Assim, os ODS surgem como um roteiro para um futuro sustentável, guiando nações ao redor do mundo na procura de soluções que equilibrem as necessidades económicas, sociais e ambientais, enfrentando simultaneamente desafios globais, desde a desigualdade social até as mudanças climáticas^(2)^.

O termo “sustentabilidade” tem raízes na palavra latina *sustentabile*, referindo-se ao estado de manter algo em certo nível, grau ou valor^(3)^. O conceito de desenvolvimento sustentável ganhou reconhecimento internacional em 1972, na Conferência das Nações Unidas sobre o Ambiente Humano em Estocolmo, que abordou o impacto prejudicial do uso indiscriminado de recursos naturais. Este conceito foi solidificado em 1983, quando a Comissão Mundial para o Ambiente e Desenvolvimento produziu o relatório Brundtland, salientando que o desenvolvimento sustentável deve atender às necessidades presentes sem comprometer a capacidade das gerações futuras de atender às suas próprias necessidades, permitindo alcançar um desenvolvimento social, económico e humano satisfatório, fazendo um uso racional dos recursos e preservando a natureza^(4)^.

A Cimeira do Rio de Janeiro, em 1992, procurou reconciliar o desenvolvimento económico com a proteção ambiental, resultando na Agenda XXI^(5)^. O conceito de desenvolvimento sustentável foi novamente destacado em 1998 durante a conferência Mundial sobre a Educação no Ensino Superior no Século XXI - Visão e Ação, que decorreu em Paris. Em 2005, a ONU declarou a década da educação para o desenvolvimento sustentável, visando mudar comportamentos e capacitar as populações para construir um mundo mais sustentável. Finalmente, em 2015, os 17 ODS foram incluídos na Agenda 2030, com o objetivo de promover a aquisição de conhecimentos e competências para o desenvolvimento sustentável. Leal-Filho et al. (2017) referem que os ODS podem constituir uma oportunidade para ultrapassar os obstáculos à sustentabilidade nas IES^(6)^. Apesar de o estudo de Aleixo et al. (2018) sugerir que as IES e a sociedade reconhecem a grande importância do desenvolvimento sustentável, este ainda não foi totalmente integrado no sistema e nas atividades institucionais^(7)^.

É importante avaliar em que medida as IES incorporaram o desenvolvimento sustentável em todas as suas atividades, que no geral seguiram uma abordagem “top-down”. Este processo começou com iniciativas planeadas pelos órgãos de gestão, envolvendo posteriormente todas as partes interessadas. Uma universidade sustentável, segundo Velazquez et al. (2006), é uma IES que, em parte ou como um todo, aborda, envolve e promove a minimização dos impactos negativos sobre o ambiente, economia, sociedade e saúde, ocorrendo por meio do cumprimento das suas funções de ensino, investigação, divulgação, parceria e gestão, contribuindo para a transição de uma sociedade com estilos de vida mais sustentáveis^(8)^.

De acordo com Ramos et al. (2015), o desenvolvimento do ensino superior para a sustentabilidade implica uma inclusão mais eficaz da educação para o desenvolvimento sustentável nos cursos. Apesar de estudos de caso documentados nos últimos anos demonstrarem mudanças nos currículos das IES, a educação para o desenvolvimento sustentável ainda não é amplamente praticada e permanece como um desafio significativo que necessita de ser cumprido^(9)^.

As IES desempenham um papel fundamental na promoção do desenvolvimento sustentável, contribuindo, desta forma, significativamente para os ODS. Essa contribuição ocorre em quatro áreas essenciais, alinhadas à missão e atuação das IES. Estas áreas incluem a condução de investigação inovadora, a oferta de ensino alinhado com princípios da sustentabilidade, a gestão ambiental responsável dos campus e a liderança ativa na promoção de práticas sustentáveis^(10)^.

O presente estudo centra-se assim no papel das IES no cumprimento dos ODS, explorando como podem integrá-los nas suas atividades diárias, tais como currículos académicos, atividades de investigação, campus e liderança. Ao considerar o compromisso institucional com os ODS, examinaremos um exemplo de uma IES, portuguesa, que tem sido catalisadora de mudanças significativas em direção a um desenvolvimento mais sustentável.

## OBJETIVO

Descrever as atividades, na área de Investigação, Ensino, *Campus* e Liderança, que visam o cumprimento dos Objetivos de Desenvolvimento Sustentável (ODS), dinamizados por uma Instituição de Ensino Superior, integrada no ensino politécnico, da região norte de Portugal.

## MÉTODOS

Trata-se de um relato de experiência de uma IES, durante o ano de 2022, no que se refere às atividades que visam o cumprimento dos 17 ODS^(10)^ nas diferentes tipologias de atividades.

### Contexto de relato de experiência

Esta IES em 2021 aderiu ao Observatório de Responsabilidade Social e Instituições de Ensino Superior (ORSIES), o que implicou a concretização do mapeamento das atividades na área de ensino e investigação que dão cumprimento aos ODS.

Esta IES, que leciona exclusivamente enfermagem, tem como visão a construção de uma enfermagem mais significativa para as pessoas. Neste sentido, estabelece um compromisso com a sociedade, a profissão e a comunidade de edificar uma Enfermagem baseada em conhecimento e fomentar a aquisição de competências que respondam aos desafios societais que o século XXI impõe (Plano estratégico 2020-2024).

De acordo com o guia “Acelerando a educação para os ODS nas universidades: um guia para universidades, faculdades e instituições de ensino superior”^(11)^, as IES podem contribuir para os ODS em quatro áreas, considerando a sua missão e intervenção, nomeadamente, na investigação, no ensino, na liderança e na gestão.

### Procedimento para recolha e organização da informação

Reconhecendo a importância das práticas de sustentabilidade, analisaram-se os indicadores que dão resposta aos ODS, através do mapeamento e classificação das atividades segundo as recomendações da SDSN Australia^(1)^. Este mapeamento foi realizado com recurso a um processo manual de leitura e interpretação da informação. Esta abordagem envolveu uma análise documental das fontes de dados e uma avaliação manual, com a respectiva atribuição de actividades aos ODS, por ser técnica e logisticamente, uma abordagem mais simples.

Para a organização da informação, os resultados mapeados por ODS, das atividades realizadas nesta IES, foram agrupados nas seguintes categorias: ensino, investigação, campus e liderança (para mais detalhes ver Figura 1). Mais especificamente, na categoria ensino estão os diferentes cursos lecionados por esta IES e as unidades curriculares (UC) do curso de licenciatura em Enfermagem (CLE). Para o devido efeito, as fontes de informação sujeitas a análise foram as fichas de UC do CLE e a informação global dos restantes cursos; na investigação mapearam-se as publicações de artigos científicos, através da leitura dos títulos e do resumos, bem como a análise dos projetos de investigação em curso; já no *campus* estão as atividades que dão “vida a um campus académico” como os eventos (exemplo congressos), notícias de eventos realizados e promovidos por esta comunidade, podcast e ainda atividades de cariz solidário; por fim, atividades de liderança como instrumentos de gestão em uso, plano estratégicos e o Quadro de Avaliação e Responsabilização (QUAR). O QUAR é um quadro referencial da avaliação de desempenho dos serviços (missão), dos seus propósitos de ação (objetivos estratégicos), das metas a alcançar, dos indicadores de desempenho e respetivas fontes de verificação, dos meios disponíveis (humanos e financeiros) e da aferição da sua concretização e da identificação sumária dos desvios e respetivas causas apurados no fim do ciclo de gestão. Os dados gerados foram analisados por meio do uso de estatística descritiva, com recurso a tabelas e gráficos para a síntese da informação.

**Figura 1:** Atividades consideradas no mapeamento por área

Legenda: *QUAR - Quadro de avaliação e responsabilização.

## RESULTADOS

A Figura 1, foi adaptada para o mapeamento por áreas (investigação, liderança, *campus* e ensino) e apresenta as atividades consideradas na organização da informação.

Em seguida apresentam-se os resultados do mapeamento, por cada ODS das atividades realizadas na IES em estudo, referentes às áreas de ensino, investigação, campus e liderança.

Na área de ensino efetuou-se a quantificação das atividades que contribuem para os ODS relativamente aos cursos e UCs, ver Tabela 1. Analogamente, identificou-se na área de investigação, a contribuição dos artigos científicos e dos projetos de investigação em desenvolvimento por ODS. Ainda, na Tabela 1, realça-se na área do *campus*, que compreende o número de eventos, notícias, podcasts e atividades realizadas de âmbito solidário, e por último, pode-se verificar as atividades realizadas no âmbito da liderança que contribuem também para os ODS.

**Tabela 1:** Quantificação total das atividades de 2022 que contribuíram para os ODS

Áreas	Atividades	1	2	3	4	5	6	7	8	9	10	11	12	13	14	15	16	17
Ensino	Cursos	1	0	33	33	0	0	0	1	33	0	0	0	0	0	0	0	0
	Unidades curriculares	10	0	34	34	0	0	0	10	1	0	0	0	0	0	0	0	0
Investigação	Artigos científicos	6	0	165	23	2	0	0	10	51	7	1	0	1	0	0	7	2
	Projetos de investigação	0	0	58	19	0	0	0	10	58	0	0	0	0	0	0	2	10
*Campus*	Eventos	2	0	8	18	1	0	0	2	1	3	1	0	0	0	0	14	13
	Notícias	5	0	32	27	0	0	0	2	11	13	2	0	3	1	1	13	39
	Podcast	0	0	18	4	0	0	0	7	0	1	1	0	0	0	0	1	1
	Atividades Solidárias	23	17	42	33	8	11	5	5	5	49	5	9	5	5	5	54	48
Liderança	Instrumentos de gestão/ Plano estratégico/QUAR	4	1	2	8	3	0	0	2	4	7	0	1	0	1	1	12	5

*Legenda: QUAR - Quadro de avaliação e responsabilização.*

Relativamente à área de ensino (cursos), na Tabela 2, assinala-se a distribuição de ODS por licenciatura, mestrados, pós-graduações e doutoramento.

**Tabela 2:** Atribuição de ODS, por cursos conferentes de grau e não conferentes de grau, em 2022

Curso/ODS	1	2	3	4	5	6	7	8	9	10	11	12	13	14	15	16	17
Licenciatura	1	0	1	1	0	0	0	1	1	0	0	0	0	0	0	0	0
Mestrados	0	0	21	21	0	0	0	0	21	0	0	0	0	0	0	0	0
Pós-graduações	0	0	10	10	0	0	0	0	10	0	0	0	0	0	0	0	0
Doutoramento	0	0	1	1	0	0	0	0	1	0	0	0	0	0	0	0	0
Total dos cursos (licenciatura, mestrado, pós-graduações e doutoramento	1	0	33	33	0	0	0	1	33	0	0	0	0	0	0	0	0

No que diz respeito aos artigos científicos, foi feita uma análise comparativa da distribuição dos ODS atribuídos manualmente e pela classificação Scopus conforme apresentado na Tabela 3. A atribuição manual dos ODS foi realizada de acordo com o conteúdo dos artigos (título e resumo) e não apenas por palavra-chave, tal como acontece na Scopus.

**Tabela 3:** Quantidade de artigos científicos distribuídos pelos ODS por classificação manual vs classificação da Scopus

Classificação	1	2	3	4	5	6	7	8	9	10	11	12	13	14	15	16	17
Manual	6	0	165	23	2	0	0	10	51	7	1	0	1	0	0	7	2
Scopus	1	0	12	0	1	0	0	1	1	2	0	0	0	0	0	0	0

A Tabela 4 agrega a distribuição total das atividades realizadas nesta IES, por ODS e 5P, destacando-se um total de 1247 atividades.

**Tabela 4:** Distribuição total das atividades de 2022 por ODS e 5P

ODS	Pessoas							Planeta				Prosperidade					Paz	Parcerias	TOTAL
	1	2	3	4	5	6	7		8	9	10	11	12	13	14	15	16	17	
Atividades/contribuições	51	18	392	199	14	11	5		49	164	80	10	10	9	7	7	103	118	1247

*ODS - Objetivos de desenvolvimento sustentável*

Os dados demonstram que a IES, de um total de 1247 atividades mapeadas e classificadas para os ODS, atribui maior preponderância à dimensão “Pessoas”, representando 55% (n= 685) das atividades classificadas. Na dimensão “Pessoas”, destaque para a centralidade do ODS 3 - Saúde e Bem-Estar - que corresponde a 32% (n= 392) do total de atividades elencadas.

Relativamente à dimensão “Planeta”, com um total de 293 atividades (24% do total de atividades), destaca-se o ODS 9 - Indústria, Inovação e infraestruturas com 164 atividades (13% do total de atividades).

Na dimensão “Prosperidade” (n=43), o ODS 11 - Cidades e Comunidades Sustentáveis e o ODS 12 - Consumo e Produção Responsáveis, apresentam 10 atividades cada, correspondendo a parte residual da amostra (3%). Por último, as dimensões “Paz” e “Parcerias” com 103 e 118 atividades, cada, representando 8% e 10% do total das atividades classificadas.

## DISCUSSÃO

Esta IES que está centrada no ensino e investigação em enfermagem, apresenta uma contribuição para os ODS na área da saúde e educação, refletindo a sua missão de formar profissionais de saúde e promover o conhecimento científico.

No âmbito do ensino, a análise quantitativa das atividades educacionais, como cursos e UCs, revela uma centralidade para os ODS 3 (Saúde e Bem-Estar), 4 (Educação de Qualidade) e 9 (Indústria, Inovação e Infraestrutura), com maior incidência no primeiro e segundo ciclos de estudos. Contudo, observa-se uma sub-representação de outros ODS importantes, que pode não corresponder à realidade do conteúdo lecionado, remetendo para a necessidade de consciencializar os docentes a intervir no processo de classificação.

No domínio da investigação, a discrepância entre as classificações automáticas, como as da Scopus (baseada no algoritmo que combina um conjunto de *keywords*), e as análises manuais indica uma tendência de subestimação da contribuição dos estudos de enfermagem para os ODS. Visto que a classificação automática parece não ter refletido o impacto dos artigos na área da saúde global e educação de qualidade, já a análise manual revela um contributo substancial, especialmente para o ODS 9 (Inovação). Portanto, tratando-se de publicações na área de Enfermagem, a contagem dos ODS segundo a Scopus, parece não considerar o contributo desta disciplina para a sustentabilidade e responsabilidade social. Além do mais, por parte desta instituição à um claro compromisso com a sustentabilidade e responsabilidade social, patenteado no seu Plano Estratégico 2020-2024, que apresenta ações voltadas para a saúde, segurança, eficiência energética e bem-estar da comunidade. A instituição também se empenha em combater o abandono escolar, bem como fomentar a empregabilidade dos seus licenciados, promovendo uma educação inclusiva e práticas que equilibram a vida pessoal, familiar e profissional.

Já as atividades solidárias e iniciativas de responsabilidade social têm dado dinâmica e significado ao campus, evidenciando o compromisso desta IES na criação de um ambiente educativo que atenda às necessidades dos estudantes, mas que também contribua para o desenvolvimento sustentável da sociedade. Deste modo, este relato de experiência reflete um trabalho de monitorização, mapeamento e classificação de atividades por ODS, através de uma abordagem holística que integra ensino, investigação e responsabilidade social, passível de ser replicável noutras IES.

## CONCLUSÕES

Em conclusão, a análise dos dados evidencia o compromisso desta IES com os ODS, em particular nas áreas de saúde, educação e inovação.

Este compromisso não se manifesta apenas nas atividades correntes, mas também, em termos de implicação para a prática, na projeção estratégica e na implementação de indicadores de sustentabilidade específicos. Nesta ótica, este trabalho tem também contribuído para a adoção e registo de indicadores como sejam o número de estudantes com apoio financeiro (ODS1), o consumo de água (ODS6), o número de patentes (ODS9), entre outros. O que reflete a sua estratégia sistematicamente integrada que se objetiva não apenas no cumprimento dos ODS, mas ainda na respetiva monitorização e avaliação contínua do impacto das ações na instituição. Esta prática facilita a autoavaliação contínua, reforça o alinhamento com o plano estratégico da instituição, assegurando que as atividades em curso e futuras se mantenham focadas na promoção do desenvolvimento sustentável. A integração destes indicadores na prática diária da IES reforça o seu papel enquanto instituição líder no ensino da enfermagem, não só na região norte de Portugal, mas também como modelo de sustentabilidade no contexto do ensino superior. Esta abordagem estratégica não apenas atende ao objetivo imediato de cumprir os ODS, bem como estabelece um caminho sustentável para o futuro, onde a educação, a investigação e a liderança se entrelaçam para promover um impacto positivo e duradouro em termos societais. Finalmente, conclui-se que há necessidade de uma maior consciencialização e formação dos docentes responsáveis pelo planeamento dos conteúdos letivos, no sentido de estruturarem as suas UC ou Cursos, tendo por base os contributos para os ODS.
